# C-reactive Protein Levels After Anterior Cruciate Ligament Reconstruction

**DOI:** 10.7759/cureus.71473

**Published:** 2024-10-14

**Authors:** Ryohei Uchida, Yuzuru Ueda, Ryo Iuchi, Rikio Takao, Takashi Kanamoto, Yoshinari Tanaka, Yoshiki Shiozaki, Shuji Horibe

**Affiliations:** 1 Department of Orthopaedic Sports Medicine, Kansai Rosai Hospital, Amagasaki, JPN; 2 Sports Medicine, Tenjin Clinic, Toyonaka, JPN; 3 Department of Nutrition, Graduate School of Human Life and Ecology, Osaka Metropolitan University, Habikino, JPN; 4 Department of Medicine for Sports and Performing Arts, Osaka University Graduate School of Medicine, Suita, JPN; 5 Department of Sports Medicine, Seifu Hospital, Sakai, JPN

**Keywords:** acl reconstruction, age, bmi, c-reactive protein, infection, postoperative complication, sex

## Abstract

Introduction

Understanding standard changes in C-reactive protein (CRP) after Anterior cruciate ligament reconstruction (ACLR) is important for early detection of septic arthritis.

Methods

We conducted a prospective two-center comparative study to clarify changes in postoperative CRP levels after ACLR, as well as factors that influence the changes, in a large sample of Asian patients. A total of 439 ACLR cases were included in this study. CRP tests were performed before and one, seven, and 14 days after ACLR.

Results

The mean CRP level was 0.06 ± 0.19 mg/dl (range: 0-2.69) preoperatively, increased to 0.54 ± 0.58 (range: 0-4.38) mg/dl at one day postoperatively and 0.92 ± 1.22 (range: 0-11.48) mg/dl at seven days postoperatively, and decreased to 0.23 ± 0.35 (range: 0-3.11) mg/dl at 14 days postoperatively. Multivariate multiple regression analysis revealed that sex, BMI, and which facility the surgery was performed significantly affect CRP levels after one day postoperatively, and sex, BMI, and age significantly affect CRP levels after seven days postoperatively. Only sex was found to significantly affect CRP levels at 14 days postoperatively.

Conclusion

The present study provides a standard change in CRP after ACLR in a sufficient number of patients without postoperative complications, which would be helpful to rule out the complications. Moreover, the multivariate analysis revealed that sex, age, BMI, and facility are factors that significantly affect CRP levels after ACLR.

## Introduction

Postoperative infection is a difficult complication to treat in orthopedic surgery, and early diagnosis is crucial. Definitive diagnosis requires the identification of causative bacteria, but since this information might not be immediately available, symptoms (e.g., fever, swelling, pain) and laboratory findings (e.g., white blood cell count, erythrocyte sedimentation rate, C-reactive protein (CRP)) are used to make an early diagnosis. Among markers of postoperative infection, CRP is considered the most sensitive because the value of CRP correlates with the intensity of the inflammatory conditions [1]. Therefore, CRP is also useful for grasping the pathology as well as the evaluation of treatment effects. Moreover, CRP is readily available in most clinical settings. However, its changes and peak levels differ by the degree of surgical invasiveness. In a previous study [2], CRP reached peak levels (mean, 4.8 mg/dl) on the second day after lumbar microdiscectomy and thereafter decreased to normal levels in 5 to 10 postoperative days. In the case of joint replacement, peak levels exceeding 10 mg/dl were noted on the second or third postoperative day, but levels remained elevated relative to preoperative levels even after two weeks (2.5 to 4.0 mg/dl) and returned to normal levels in 21 to 42 postoperative days [2,3]. As such, both peak levels and changes in postoperative CRP vary depending on the type of surgery. Thus, understanding standard CRP changes and peaks for each surgical procedure is important for the early diagnosis of postoperative infection. 

Anterior cruciate ligament reconstruction (ACLR) is one of the most commonly performed orthopedic surgeries [4,5]. Patients who undergo ACLR are mainly young people aiming to return to sports, and the outcomes are relatively favorable [6,7]. However, serious postoperative complications such as infection, deep vein thrombosis, and pulmonary embolism have also been reported [8-14]. In particular, septic arthritis has a relatively high incidence of 0.25-1.5% [8,12,13], and there is concern that its onset not only renders treatment difficult but also significantly affects the patient's ability to return to sports (i.e., the main objective of the surgery) [15]. Hence, understanding standard changes in CRP after ACLR, as well as other orthopedic surgeries, is also important from the standpoint of early detection of septic arthritis. According to previous reports [1,16,17], CRP levels peak at three to seven days postoperatively, with a mean value ranging from 1.0 to 3.7 mg/dl. Changes in CRP are reportedly affected by various factors, including sex, race, and age. However, due to the small number of cases and lack of comparative multi-center studies, these factors are not fully understood. Moreover, no study has been conducted on Asian populations.

Against this backdrop, we conducted a prospective two-center comparative study to clarify changes in postoperative CRP levels after ACLR, as well as factors that influence the changes, in a large sample of Asian patients.

## Materials and methods

Subjects

Subjects were patients who underwent ACLR with a hamstring tendon (HT) graft or bone-patellar tendon-bone (BTB) graft under arthroscopy at two facilities (Seifu Hospital, Sakai, Japan (Hospital S) and Osaka Rosai Hospital, Sakai, Japan (Hospital O)) between April 2012 and May 2017. After excluding patients with complex ligament injuries and liver disease and those with missing data, 439 (215 males, 224 females; mean age, 24.2 ± 9.9 years) were included in this study. Hospital S is a medium-sized hospital with about 200 beds, while Hospital O is a large hospital with about 700 beds; the same clinical path for anterior cruciate ligament (ACL) reconstruction, perioperative antibiotics and medications, and postoperative rehabilitation were the same in both facilities, Hospital S and Hospital O. The facilities are located close to each other, and also the structure of the operating rooms is similar. Although the difference regarding the infection control team was unknown, the number of surgeons differed. In April 2012, there were two surgeons with 11 and 31 years of experience in Hospital S, while there were four surgeons with 11, 12, 13, and 18 years of experience in Hospital O. 

Ethical statement

This study was approved by the Ethics Committee of Seifu Hospital, Sakai, Japan (Approval number: 23-1) and was conducted in accordance with the principles set forth in the Helsinki Declaration. All patients received an explanation regarding the use of data for study purposes and provided written consent to participate in the study before undergoing surgery. If the patient was a minor, consent from the patient's parent or guardian was obtained.

Operative procedures and postoperative regimen

Anatomic double-bundle or triple-bundle ACLR with an autologous HT graft was performed as follows [18,19]. After harvesting the semitendinosus tendon, femoral and tibial tunnels were anatomically created under arthroscopy. The tendon graft was then passed through the tunnels and fixed with Endbutton-CL on the femoral side and a double spike plate on the tibial side. 

Anatomic rectangular tunnel ACLR with an autologous BTB graft was performed as follows [20-22]. After harvesting the BTB graft, rectangular femoral and tibial tunnels were anatomically created under arthroscopy. The tendon graft was then passed through the tunnels and fixed with double spike plates on both the femoral and tibial sides. 

To prevent infection, cefmetazole sodium 2g per day in two divided doses was administered intravenously for three days from the day of surgery. For the postoperative pain management, total 180mg of loxoprofen sodium was taken in three divided doses per day for one week after operation. In patients who suffered from pain, the drug was sometimes administered as an occasional dose or as an additional dose after the first postoperative week, but most patients did not take painkillers. During their hospitalization, icing of the surgical site was performed on all patients. 

Postoperative rehabilitation included a range of motion training, which was initiated after two weeks of orthotic immobilization. Patients were allowed to bear partial weight at three weeks postoperatively. Full weight bearing was started at four weeks postoperatively. CRP tests were performed using the latex agglutination method before one, seven, and 14 days after ACLR. The lower detection limit was 0.05 mg/dl. CRP levels below this limit were defined as 0 mg/dl. These surgical procedures and postoperative protocol (medication, icing, rehabilitation, and blood tests) were the same in all patients regardless of facilities.

Statistical analysis

Data are shown as mean ± standard deviation. All statistical analyses were performed using IBM SPSS software, version 23 (IBM Corp., Armonk, NY). P<0.05 was considered statistically significant. Independent variables included in the analyses were sex, age, body mass index (BMI), type of surgery (primary or revision), additional meniscus surgery (resection or repair), additional cartilage surgery (articular cartilage transplantation or chondral microfracture), type of graft, which facility the surgery was performed (Hospital S or Hospital O), and surgeon experience. 

## Results

Table 1 shows epidemiological and surgical data for all patients. In total, 391 patients underwent primary arthroscopic ACLR, and 48 underwent revision arthroscopic ACLR. Autologous HT grafts were used in 209 cases (average age: 25.0 ± 12.0), and BTB grafts were used in 230 (average age: 23.4 ± 7.6). There were 74 cases of meniscal resection, 139 cases of meniscal repair, three cases of chondral microfracture, and two cases of articular cartilage transplantation. Surgeries were performed at Hospital S in 155 cases and Hospital O in 284 cases. Mean number of years of experience among surgeons was 20.0 (range, 11-38) years. There was no complication in this study.

**Table 1:** Epidemiological and surgical data for all patients ACLR: anterior cruciate ligament reconstruction, HT: hamstring tendon, BTB: bone-patellar tendon-bone, MF: chondral microfracture, ACT: articular cartilage transplantation; Hospital S: Seifu Hospital, Sakai, Japan; Hospital O: Osaka Rosai Hospital, Sakai, Japan.

Total number	439
Age	24.0±9.9
Sex (Male/Female)	215/224
Type of surgery (Primary ACLR/Revision ACLR)	391/48
Type of graft (HT/BTB)	209/230
Additional procedures(Meniscal resection/Meniscal repair/MF/ACT)	74/139/3/2
Facility (Hospital S/Hospital O)	155/284
Surgeon experience (number of years)	20.0 (11-38)

**Figure 1:**
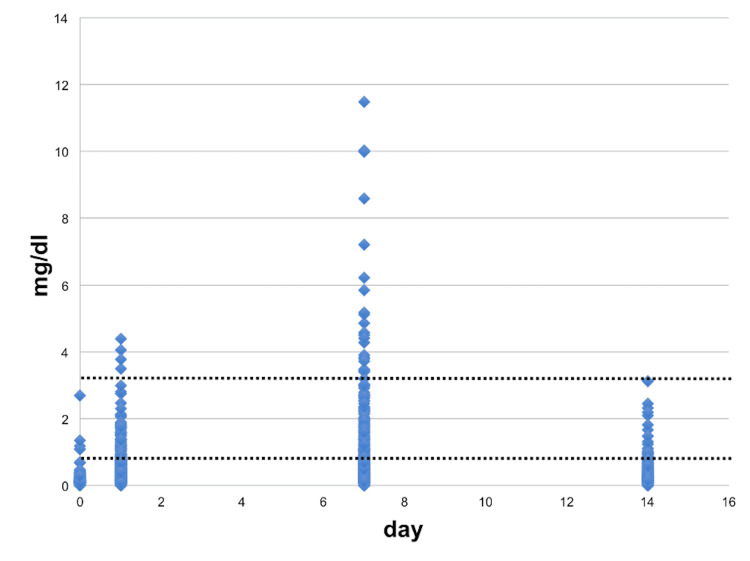
Scatter diagram of pre- and postoperative C-reactive protein levels

CRP data for all patients is shown in Figure 1. The number of patients with greater than normal CRP levels (normally defined as <0.3 mg/dl [23]) was 21 (4.7%) preoperatively, and 253 (57.6%), 307 (69.9%), and 94 (21.4%) at one, seven, and 14 days postoperatively, respectively.

**Figure 2:**
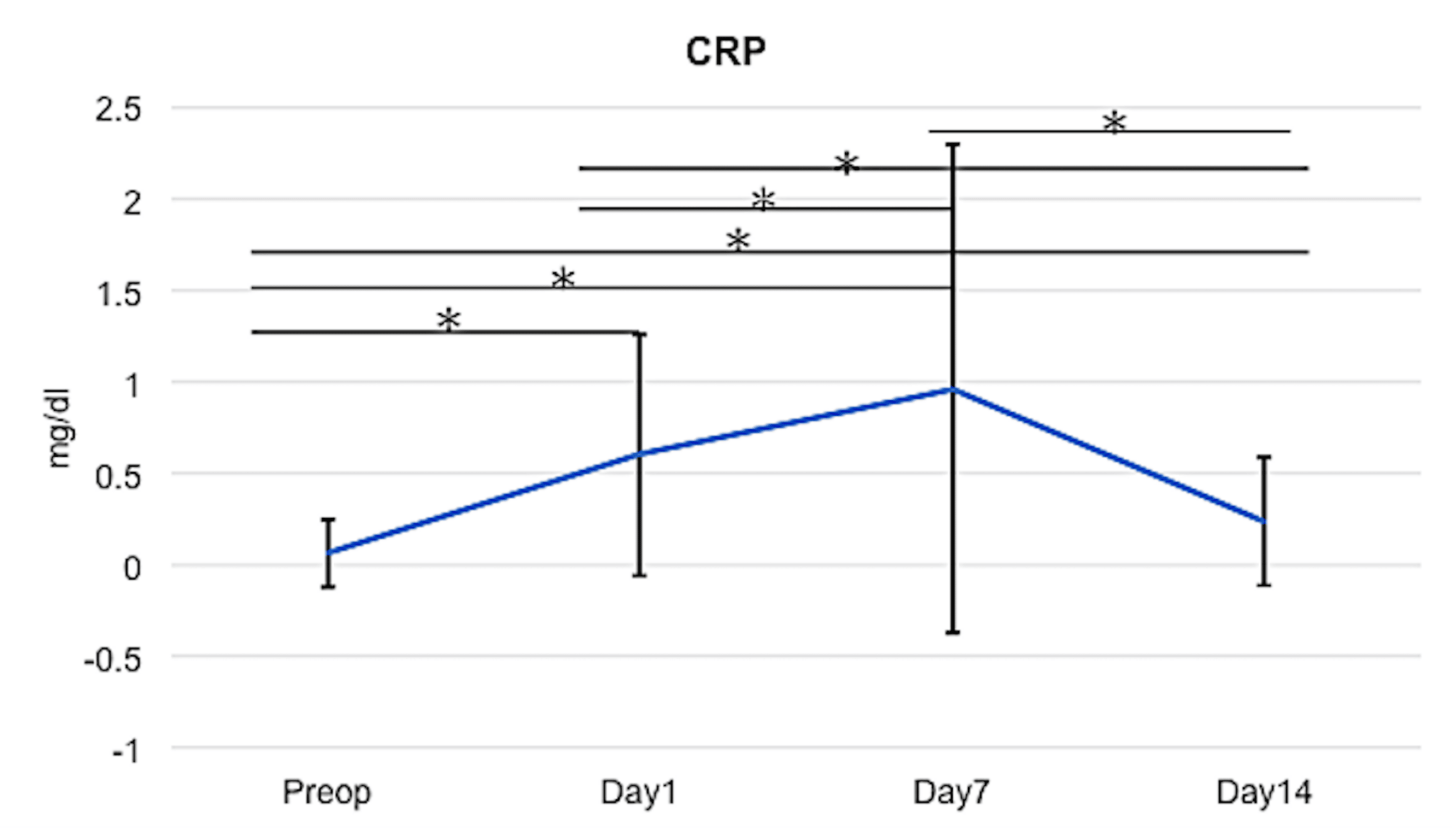
Changes in mean C-reactive protein levels ANOVA Tukey-Kramer test, *p＜0.05

Changes in mean CRP levels are shown in Figure 2. The mean CRP level was 0.06 ± 0.19 mg/dl (range: 0 - 2.69) preoperatively, increased to 0.54 ± 0.58 (range: 0-4.38) mg/dl at one day postoperatively and 0.92 ± 1.22 (range: 0-11.48) mg/dl at seven days postoperatively, and decreased to 0.23 ± 0.35 (range: 0-3.11) mg/dl at 14 days postoperatively. Mean postoperative CRP levels were all significantly higher compared to the mean preoperative CRP level (p<0.05).

Multivariate multiple regression analysis revealed that sex, BMI, and facility significantly affect CRP levels at one day postoperatively (Table 2), and sex, BMI, and age significantly affect CRP levels at seven days postoperatively (Table 3). Only sex was found to significantly affect CRP levels at 14 days postoperatively (Table 4).

**Table 2:** Results of multivariate analysis (postoperative CRP levels at one day postoperatively) ACLR: anterior cruciate ligament reconstruction, HT: hamstring tendon, BTB: bone-patellar tendon-bone; BMI: body mass index; SE: standard error; CI: confidence interval; CRP: C-reactive protein ^a ^The mean CRP level in male was significantly higher than that in female. ^b ^The mean CRP level in patients in Hospital S was significantly higher than that in Hospital O.

	SE	β	95%CI	p
Sex^a^	0.059	0.164	0.083 to 0.314	0.001
Age	0.003	0.000	-0.005 to 0.005	0.999
BMI	0.005	0.320	0.024 to 0.042	<0.001
Type of surgery (Primary ACLR or Revision ACLR)	0.089	0.055	-0.068 to 0.281	0.231
Additional procedures				
Meniscal resection	0.071	-0.016	-0.165 to 0.114	0.715
Meniscal repair	0.057	0.079	-0.010 to 0.215	0.075
Chondral microfracture	0.314	-0.007	-0.668 to 0.564	0.868
Articular cartilage transplantation	0.386	0.048	-0.330 to 1.187	0.267
Type of graft (HT or BTB)	0.059	0.022	-0.089 to 0.142	0.652
Facility (Hospital S or Hospital O)^b^	0.076	-0.257	-0.474 to -0.176	<0.001
Surgeon experience	0.004	-0.061	-0.012 to 0.004	0.311

**Table 3:** Results of multivariate analysis (postoperative CRP levels at seven days postoperatively) ACLR: anterior cruciate ligament reconstruction, HT: hamstring tendon, BTB: bone-patellar tendon-bone; BMI: body mass index; SE: standard error; CI: confidence interval; CRP: C-reactive protein; Hospital S: Seifu Hospital, Sakai, Japan; Hospital O: Osaka Rosai Hospital, Sakai, Japan. ^a^ The mean CRP level in males was significantly higher than that in females.

	SE	β	95%CI	p
Sex^a^	0.126	0.316	0.525 to 1.020	<0.001
Age	0.006	-0.106	-0.025 to -0.002	0.027
BMI	0.010	0.113	0.004 to 0.043	0.017
Type of surgery (Primary ACLR or Revision ACLR)	0.190	-0.045	-0.551 to 0.197	0.354
Additional procedures				
Meniscal resection	0.152	-0.045	-0.445 to 0.53	0.336
Meniscal repair	0.123	0.021	-0.186 to 0.297	0.652
Chondral microfracture	0.671	0.027	-1.714 to 0.925	0.557
Articular cartilage transplantation	0.826	-0.009	-1.795 to 1.454	0.836
Type of graft (HT or BTB)	0.126	-0.013	-0.279 to 0.218	0.809
Facility (Hospital S or Hospital O)	0.162	0.010	-0.293 to 0.345	0.874
Surgeon experience	0.009	0.065	-0.008 to 0.026	0.309

**Table 4:** Results of multivariate analysis (postoperative CRP levels at 14 days postoperatively) ACLR: anterior cruciate ligament reconstruction, HT: hamstring tendon, BTB: bone-patellar tendon-bone; BMI: body mass index; SE: standard error; CI: confidence interval; CRP: C-reactive protein; Hospital S: Seifu Hospital, Sakai, Japan; Hospital O: Osaka Rosai Hospital, Sakai, Japan. ^a^ The mean CRP level in male was significantly higher than that in female.

	SE	β	95%CI	p
Sex^a^	0.036	0.304	0.140 to 0.282	0.000
Age	0.002	0.027	-0.002 to 0.004	0.581
BMI	0.003	0.049	-0.003 to 0.008	0.300
Type of surgery (Primary ACLR or Revision ACLR)	0.054	-0.064	-0.178 to 0.036	0.190
Additional procedures				
Meniscal resection	0.043	0.048	-0.041 to -0.130	0.306
Meniscal repair	0.035	0.066	-0.020 to 0.118	0.163
Chondral microfracture	0.192	-0.016	-0.043 to 0.312	0.734
Articular cartilage transplantation	0.236	-0.021	-0.572 to 0.357	0.650
Type of graft (HT or BTB)	0.036	-0.043	-0.101 to 0.041	0.405
Facility (Hospital S or Hospital O)	0.046	0.101	-0.018 to 0.165	0.114
Surgeon experience	0.003	0.049	-0.003 to 0.007	0.446

## Discussion

The main findings of this study were 1) CRP levels reached a peak level of 0.92 ± 1.22 mg/dl at seven days postoperatively in ACLR patients with no postoperative infection complications, with a subsequent decrease noted at 14 days postoperatively; 2) sex, patient age, BMI, and facility where surgery was performed were factors that affected postoperative CRP levels; and 3) mean CRP levels in the present study were lower compared to those reported previously in Europe and the United States [1,16,17].

The sensitivity of CRP as a marker for infection has been reported to be the highest among all parameters [24]. However, as reported previously, peak CRP levels after ACLR differed from those observed after total knee arthroplasty (i.e., roughly 5-fold higher). Thus, understanding standard changes in CRP according to the type of surgery is important. When CRP levels are elevated only slightly, as in the case of post-ACLR patients, it is sometimes difficult to judge whether the increase is due to surgical invasion or postoperative infection. Previous studies reported that CRP levels increased to ≥10 mg/dl on the 14th postoperative day in patients with post-ACLR infection, whereas in those with no complications, the levels remained below 5 mg/dl [16,25]. In the present series as well, CRP levels at 14 days postoperatively did not exceed 5 mg/dl in all cases. When we further examined patients with high postoperative CRP (defined as ≥3.0 mg/dl), only one patient was identified as having high CRP at 14 days postoperatively, whereas four and 24 patients were identified at one and seven days postoperatively, respectively. These data suggest the need for an early response if CRP levels exceed 3 mg/dl on postoperative day 14, keeping in mind the possibility that the patient might have developed postoperative infection. 　

Among the various factors affecting CRP and the course of its changes after ACLR, male sex, patient age, BMI, and surgical facility were identified as significant factors. These findings are consistent with a previous report by Ruiz-Ibán et al., in which CRP levels were significantly higher among male patients [16]. This is likely attributable to differences in knee size between males and females, which may give rise to differences in the degree of surgical invasiveness. Patients with high BMI also had significantly higher CRP levels at one and seven days postoperatively, possibly due to the inflammatory effect of arachidonic acid present in fat. Furthermore, younger patients showed significantly higher CRP levels at seven days postoperatively, suggesting that changes in CRP in response to surgical invasion might be affected by age. Indeed, while the details are still unknown, age has been reported to affect CRP levels in healthy individuals [26]. Meanwhile, no significant difference was observed by graft type, contrary to a report by Calvisi et al. that postoperative CRP levels tended to be higher with BTB grafts than with semitendinosus tendon grafts [17]. Differences in operative methods or the size of harvested bones might have contributed to this discrepancy. Patients in the present series underwent ACLR at two facilities using the same operative methods, postoperative regimens, and infection measures (e.g., cleaning methods for surgical devices). Yet, comparisons between the two facilities revealed a significant difference in CRP levels (only at one day postoperatively), suggesting the possibility that differences in the operating room environment may affect CRP changes. However, changes in CRP or peak levels did not significantly differ between the two facilities. These findings suggest that changes in CRP are potentially useful for screening patients with complications such as infection. Facilities with greater changes in CRP, or higher peak levels, than those observed in the present study, may need to verify their infection control measures, operating room filter specifications, ventilation frequency, temperature, humidity, number of staff in the operating room, and washing methods for loan instruments [27].　

Limitations

This study has several limitations. First, blood collection was performed only at one, seven, and 14 days postoperatively. While our findings were consistent with those reported by Ruiz-Ibán et al. [16] (i.e., CRP reached peak levels seven days after ACLR), other studies have reported a decrease in CRP levels from three to seven days postoperatively, with a peak reached at three days postoperatively [1,17]. Increases in CRP occur due to the promotion of CRP production in the liver by inflammatory cytokines, which are produced in response to surgical invasion [23]. CRP levels peak 48 hours after physical invasion, with a half-life of 19 hours [23]. Thus, if blood tests were performed three days postoperatively, the CRP levels may have been higher (i.e., peak CRP levels at three days postoperatively). However, according to a previous report [28], post-ACLR infection is commonly observed between seven and 14 days postoperatively, indicating that it is too early to judge the presence of infection based on CRP levels at three days postoperatively. For these reasons, we considered it more appropriate to perform blood tests at seven days postoperatively. Second, since patients in the present series did not include those with complications such as postoperative infection, clinical data were not compared between patients with and without postoperative complications. However, the results of this study could be helpful in ruling out the postoperative complications including septic arthritis, because of a sufficient number of patients without postoperative complications. Moreover, the results in this study could be potentially more broadly applied by expanding materials, such as different races and age groups, in addition to improving the evaluation methods, such as the other data from blood test or simpler tests other than blood tests.

## Conclusions

The present study provides a standard change in CRP after ACLR in a sufficient number of patients without postoperative complications, which would be helpful in ruling out the complications. Moreover, the multivariate analysis revealed that sex, age, BMI, and hospital are factors that significantly affect CRP levels after ACLR.
